# Exploring the impact of pathogenic microbiome in orthopedic diseases: machine learning and deep learning approaches

**DOI:** 10.3389/fcimb.2024.1380136

**Published:** 2024-04-03

**Authors:** Zhuce Shao, Huanshen Gao, Benlong Wang, Shenqi Zhang

**Affiliations:** Department of Joint and Sports Medicine, Zaozhuang Municipal Hospital, Affiliated to Jining Medical University, Zaozhuang, China

**Keywords:** pathogenic microbiome, orthopedic, machine learning, deep learning, applications, individuation, osteoporosis, arthritis

## Abstract

Osteoporosis, arthritis, and fractures are examples of orthopedic illnesses that not only significantly impair patients’ quality of life but also complicate and raise the expense of therapy. It has been discovered in recent years that the pathophysiology of orthopedic disorders is significantly influenced by the microbiota. By employing machine learning and deep learning techniques to conduct a thorough analysis of the disease-causing microbiome, we can enhance our comprehension of the pathophysiology of many illnesses and expedite the creation of novel treatment approaches. Today’s science is undergoing a revolution because to the introduction of machine learning and deep learning technologies, and the field of biomedical research is no exception. The genesis, course, and management of orthopedic disorders are significantly influenced by pathogenic microbes. Orthopedic infection diagnosis and treatment are made more difficult by the lengthy and imprecise nature of traditional microbial detection and characterization techniques. These cutting-edge analytical techniques are offering previously unheard-of insights into the intricate relationships between orthopedic health and pathogenic microbes, opening up previously unimaginable possibilities for illness diagnosis, treatment, and prevention. The goal of biomedical research has always been to improve diagnostic and treatment methods while also gaining a deeper knowledge of the processes behind the onset and development of disease. Although traditional biomedical research methodologies have demonstrated certain limits throughout time, they nevertheless rely heavily on experimental data and expertise. This is the area in which deep learning and machine learning approaches excel. The advancements in machine learning (ML) and deep learning (DL) methodologies have enabled us to examine vast quantities of data and unveil intricate connections between microorganisms and orthopedic disorders. The importance of ML and DL in detecting, categorizing, and forecasting harmful microorganisms in orthopedic infectious illnesses is reviewed in this work.

## Introduction

Orthopedic problems, such as osteoporosis, arthritis, and fractures, are becoming a more significant public health concern as the world’s population ages, these illnesses not only significantly lower patients’ quality of life on a daily basis, but they also significantly increase the financial strain on the healthcare system (Safiri et al., 2020; Steinmetz et al., 2023). The involvement of pathogenic microorganisms in the onset and progression of orthopedic illnesses has come to the attention of scientists in recent years. Research has indicated that some bacteria and viruses have the ability to directly impact bone health and accelerate the course of illness. However, classic biological techniques struggle to disclose the complex and diverse processes underlying pathogen-host interactions.

Researchers are focusing more and more on the role that pathogenic microorganisms play in orthopedic diseases because they can even directly infect orthopedic patients, such those who have fractures. Improvements in microbial detection technology have also led to an increase in the number of organisms at the site of Periprosthetic Joint Infections (PJIs) (Anagnostakos and Fink, 2021).

Machine learning and deep learning are novel methods that have gradually emerging in recent years. Many researchers have described the concepts, applications or application fields of machine learning and deep learning (Goodfellow et al., 2016; Shinde and Shah, 2018; Campesato, 2020; Janiesch et al., 2021).

In this regard, new research instruments have been made available by the emergence of machine learning and deep learning methodologies. By analyzing vast quantities of intricate biological data, spotting possible biomarkers, and forecasting illness patterns, these technologies can shed light on the connection between infections and orthopedic disorders. For instance, machine learning algorithms can predict the relationship between particular bacteria and osteoporosis by examining the genetic information and microbiological makeup of a patient. Convolutional neural networks and other deep learning techniques have been used to automatically identify lesions in imaging pictures to aid in the diagnosis of orthopedic diseases, including fractures.

Even while deep learning and machine learning have made some initial strides in this area, they still face several obstacles. There are still issues to be resolved about the interpretability of models, the quantity and quality of data restrictions, and the efficient integration of these technologies into clinical practice. Furthermore, the effective use of these technologies depends on multidisciplinary cooperation that brings together the knowledge of biologists, computer scientists, and medical professionals.

The objective of this study is to incorporate the most recent findings about the involvement of pathogenic microorganisms in orthopedic disorders, with a specific focus on the use of machine learning and deep learning techniques to enhance disease prevention, diagnosis, and therapy. We intend to give patients hope by evaluating the most recent study findings and offering recommendations for future research initiatives.

Techniques based on data, such as machine learning and deep learning, can manage vast amounts of biomedical data, including genetic, protein, and clinical data. The advent of these sophisticated methods has given scholars access to useful resources (Jordan and Mitchell, 2015; LeCun et al., 2015). To improve our comprehension of biological processes and our ability to employ data mining techniques for clinical prediction, image analysis, drug development, and other purposes, patterns and relationships in the data can be found (Rajkomar et al., 2019). Furthermore, machine learning algorithms may uncover genetic variations linked to certain diseases by examining genomic data, which aids researchers in identifying possible treatment targets.

## Standing on the shoulders of the “giants” of machine learning and deep learning for future deeper analysis of the impact of microbes on osteoporosis

Osteoporosis is a diagnosable and treatable disease, a systemic bone disease due to a decrease in bone density and bone mass and destruction of the bone microstructure due to a variety of causes, resulting in increased bone brittleness and thus susceptibility to fracture, and the concept of osteoporosis has been addressed in many studies (Schapira and Schapira, 1992; Raisz, 2005; Marcus and Bouxsein, 2008; Akkawi and Zmerly, 2018).

An international public health concern, osteoporosis is becoming more common as the world’s population ages. Patients’ quality of life is significantly impacted by the condition, which makes bones brittle and increases the chance of fractures. In spite of the fact that a variety of variables, including heredity, poor diet, and inactivity, are linked to the development of osteoporosis (Riggs and Melton Iii, 1995; Clynes et al., 2020; Salari et al., 2021), new studies have indicated that pathogenic microbes may also be a significant contributing factor (Hao et al., 2019; De Martinis et al., 2020; Cronin et al., 2022).

Pathogenic microorganisms, especially bacteria and viruses, may accelerate the process of bone loss and structural damage. Due to the complexity of pathogen host interactions and the limitations of traditional biological research methods in managing large-scale biomedical data, the exact relationship and mechanism of action between infection and osteoporosis are still unclear. This is the benefits and significance of machine learning and deep learning. With the advancement of these technologies, new tools have emerged to solve these problems, extracting patterns and features from a large amount of complex data, providing new insights into the relationship between osteoporosis and pathogenic microorganisms. For example, by using machine learning algorithms to analyze a large amount of data on the host genome, microbiome, and proteome, future research may focus on identifying microbial biomarkers associated with high risk of osteoporosis. The composition and function of the gut microbiome have a significant impact on bone metabolism, providing new ideas for the prevention and treatment of osteoporosis. Machine learning and deep learning provide more future possibilities for research in this field.

Pathogenic microorganisms have a high correlation with several established modifiable variables, which in turn function as mediators to further impact bone accrual, maintenance, and decline in the geriatric population. Preliminary research on the connection between microbes and bone health has shown that microbes can affect how interactions between bone metabolism occur, and that the gut microbiome plays a role in the regulation of bone metabolism, osteoporosis pathogenesis, prevention, and treatment (Hernandez, 2017; Ding et al., 2020; Seely et al., 2021; Guan et al., 2022).

With the rapid development of machine learning and deep learning in recent years, new perspectives have been provided to study the relationship between pathogenic microorganisms and osteoporosis. Several studies have been conducted in related fields, for example, many studies have utilized machine learning or deep learning on osteoporosis fracture rates or bioinformatics involving osteoporosis (Engels et al., 2020; Ulivieri et al., 2021; Liu et al., 2022). Most of these studies do not incorporate microbial information, which has been a limitation of previous researchers, and as more and more studies demonstrate the significant effects of certain microbes on osteoporosis, there is a need to incorporate microbial data into subsequent studies in order to obtain more rigorous and scientific results.

In order to detect osteoporosis signals in imaging data, deep learning techniques like Convolutional Neural Networks (CNNs) have made great strides in the field of medical image processing. This information may then be utilized to forecast the course of the illness and evaluate the effectiveness of treatment. Furthermore, deep learning may be utilized to combine data from other biological data sources, such microbiome data and clinical characteristics, to increase the precision of osteoporosis progression prediction.

**Figure 1:**
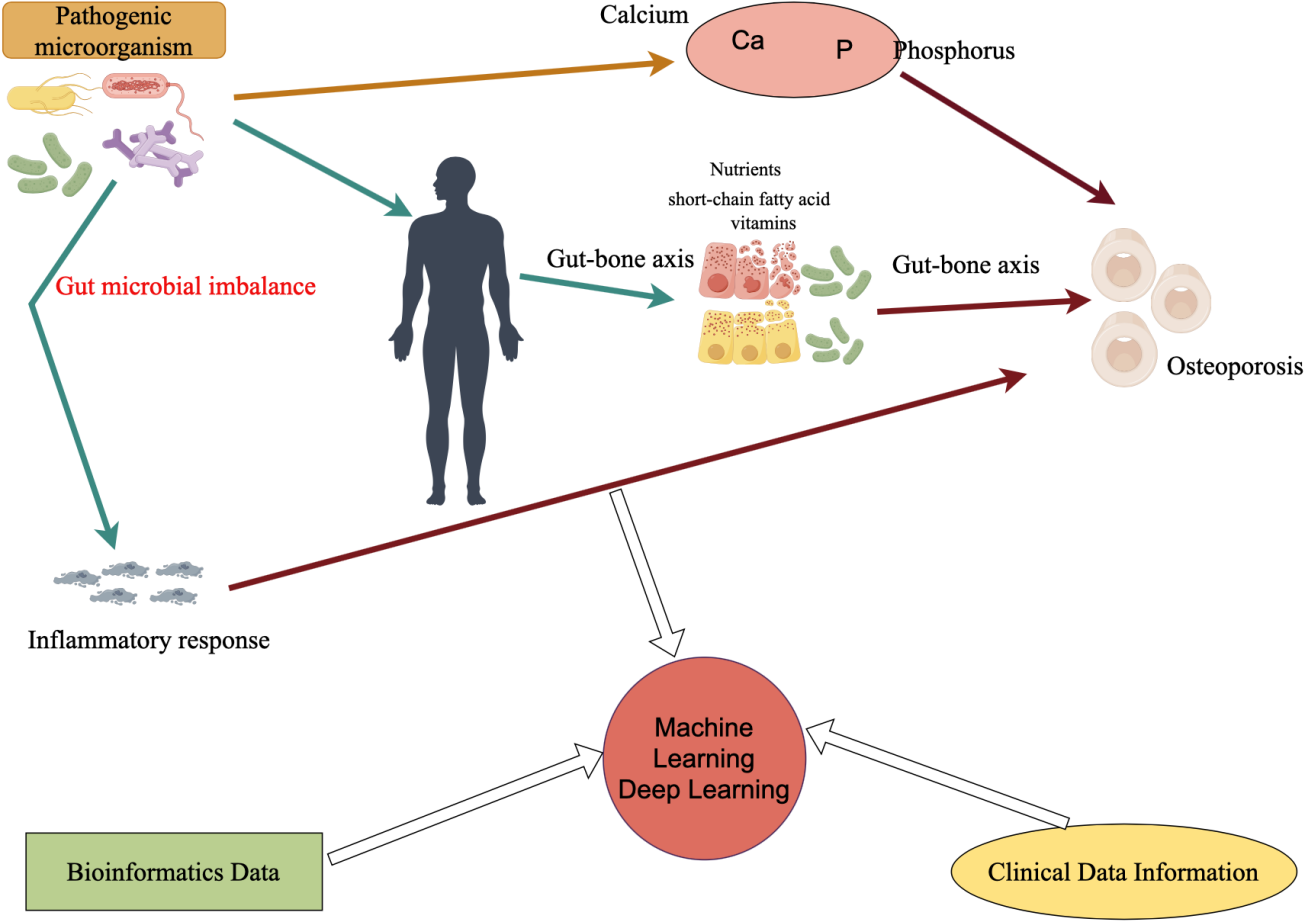
Microbes affect osteoporosis and how deep learning or machine learning can be utilized in the future. Ca refers to calcium, P refers to phosphorus, and microorganisms directly or indirectly (such as inflammatory reactions) affect the occurrence of osteoporosis.

**Figure 1** shows how pathogenic microorganisms, especially intestinal flora, can indirectly affect bone metabolism and bone mineral density through inflammatory responses or by affecting calcium and phosphorus absorption, or through the gut-bone axis, which has been found to affect the immune system, the metabolites produced (e.g., short-chain fatty acids), and by affecting the absorption of nutrients. In the future, in the study of microbial influence on osteoporosis, the addition of already mature clinical data information and bioinformatic data, and then through the powerful medium of machine learning and deep learning, will certainly bring new life and opportunities for research in related fields.

## Visualization of keywords in the field of pathogenic microorganisms using machine learning or deep learning, and a brief overview of related orthopedic diseases affected by microorganisms

In order to have a better analysis of the use of machine learning and deep learning methods to study the impact of pathogenic microorganisms in orthopedic diseases, we have attempted to provide an intuitive visualization of how researchers have used machine learning and deep learning methods to study pathogenic microorganisms in recent years, and we have utilized visualization tools to demonstrate this with the aim of getting a better grasp of the direction of development of studies exploring the impact of pathogenic microbiomes in orthopedic diseases using the premise of machine learning and deep learning methods, or an outlook on the frontiers of the future.

**Table 1** shows the top 10 keywords in terms of frequency of occurrence of machine learning and deep learning methods for research on pathogenic microorganisms, and it can be clearly found that “Artificial intelligence”, “Prediction”, “Antimicrobial resistance” are the key focus of research in this field, and also the possible future research of orthopedic microorganisms with the support of machine learning or deep learning methods.

**Table 1:** Top 10 keywords with the highest frequency of occurrence in studies analyzing pathogenic microorganisms using machine learning and deep learning methods.

ID	Keyword	Occurrences	Total link strength
1	Machine learning	104	101
2	Identification	28	53
3	Deep learning	26	33
4	Microbiology	24	42
5	Artificial intelligence	23	51
6	Classification	23	37
7	Prediction	23	53
8	Bacteria	17	22
9	Antimicrobial resistance	15	24
10	Clinical microbiology	13	18

**Figure 2:**
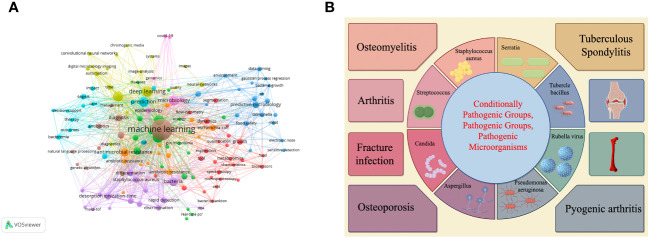
**(A, B)** Respectively demonstrate the use of machine learning or deep learning to visualize keywords in the field of pathogenic microorganisms and provide a brief overview of the types of orthopedic diseases affected by microorganisms.

We selected the WoS database on January 10, 2024 for an intuitive visual analysis of keywords for research in this area. **Figure 2A** is a collaborative linkage diagram of keywords that appear more frequently in the research of pathogenic microorganisms analyzed using machine learning and deep learning methods from 2000 to 2024, which can well show the relationship between keywords in this research field, the larger the circle indicates that this keyword plays a greater role in the research of this field, the higher the frequency of appearance, and the more the linkage between keywords indicates that the more the keyword is used in the research of this field, the more the linkage between keywords indicates that the keyword is used in the research of this field. the closer the connection between them. **Figure 2B** shows which orthopedic related diseases can be affected by microorganisms.

Visualization analysis of keywords related to research in the field of machine learning or deep learning and pathogenic microorganisms can help us identify the current focus of attention among researchers in this field and explore potential future research hotspots in this field. For example, we can see that this field seems to be closely related to “ therapy”, “expression”, “diagnosis”, etc., which may also indicate the direction of future development.

## Pathogenic microorganisms associated with orthopedic diseases

The most prevalent pathogens in orthopedic disorders are bacteria. The most frequent cause of infections, both hospital- and community-acquired, is Staphylococcus aureus. Furthermore, gram-negative bacteria like Escherichia coli and streptococci are frequently the cause of an event. Staphylococcus aureus-caused osteomyelitis is a dangerous bone infection that can result in bone death. Surgery and/or long-term antibiotic therapy are typically needed to treat this illness.

Although they are less prevalent in orthopedic diseases, fungal infections can nevertheless pose a major risk to individuals with weakened immune systems. Aspergillus and Candida are common fungi that cause problems. Patients with weakened immune systems, such those receiving organ transplants or HIV, are more likely to get fungal infections. Antifungal drugs are typically needed to treat these infections.

Even though measles and rubella viruses are not common, they can still impact bone health. This is the case with viral bone disease. The goal of treating viral bone disease is to improve the immune system’s capacity to combat the virus while also managing its symptoms.

Machine learning and deep learning techniques have become valuable tools in orthopedic disease research and therapy, particularly in the understanding and management of infections caused by pathogenic microorganisms. Large volumes of biological data may be processed and analyzed using these sophisticated computational techniques to provide information on pathogen features, infection patterns, illness development, and patient response to therapy.

## Identification and categorization of pathogens

Researchers can precisely detect and categorize microorganisms that cause orthopedic illnesses from clinical samples by utilizing deep learning algorithms (Bernard et al., 2022; Hu et al., 2023). This includes common bacteria like Staphylococcus aureus and other Gram-negative bacteria like Streptococcus and Escherichia coli. Physicians are able to promptly adopt focused treatment measures, such choosing the appropriate medications to fight certain bacterial illnesses, by precisely and swiftly detecting these organisms. Some studies have used machine learning or deep learning to analyze infections that cause orthopedic diseases, and some of these infections also include factors associated with pathogenic microorganisms (Martínez-Pastor et al., 2009; Goswami et al., 2022).

## Forecasting patterns of infection

Additionally, particular pathogen infection patterns, including hospital and community transmission, may be predicted using machine learning algorithms. By examining past infection data and patient characteristics, these models forecast infection outbreaks, assisting hospital management and public health officials in taking proactive steps to stop the spread of diseases (Genevès et al., 2018; Sharma et al., 2018; Teeple et al., 2020).

## Analysis of drug susceptibility

The most effective medications for Staphylococcus aureus-caused severe osteomyelitis can be identified with the use of machine learning techniques. Individualized treatment plans can be created to increase treatment effectiveness while lowering the emergence of antibiotic resistance by evaluating the pathogen’s susceptibility to various antibiotics.

**Figure 2B** shows the impact of different microorganisms on different orthopedic diseases and the utilization of deep learning or machine learning.

## Pathogenic microorganisms and arthritis

Arthritis is a group of diseases that affect millions of people worldwide and are characterized by joint inflammation, pain and dysfunction. Although the exact causes of arthritis are varied and include genetics, immune system dysregulation and environmental factors, there is growing evidence that pathogenic microorganisms, such bacteria and viruses, also play an important role in the development and progression of arthritis (Li et al., 2013; Bo et al., 2020; Berthelot et al., 2021). Arthritis is a common inflammatory joint disease involving one or more joints. Its main symptoms include joint pain, swelling, stiffness and limited movement. Depending on the cause and presentation, arthritis can be categorized into a number of types, the two most common of which are osteoarthritis (OA) and rheumatoid arthritis (RA), which puts a huge financial strain on the world’s arthritis sufferers (March and Bachmeier, 1997; Leifer et al., 2022). While the causes of arthritis are diverse, there is growing evidence that pathogenic microorganisms play a key role in many types of arthritis. It is difficult to determine the precise microbe that causes arthritis, though, as it requires removing and evaluating a lot of data from intricate biological samples. Recent years have seen a considerable advancement in the field of biomedical research, particularly in the areas of pathogen identification, genomes analysis, and illness prediction, thanks to the fast adoption of machine learning and deep learning techniques. These technological advancements provide novel approaches to the diagnosis and treatment of arthritis by processing and analyzing vast amounts of biological data, recognizing disease patterns, and forecasting the relationship between infections and illnesses. In the future, patients can receive individualized diagnoses and treatment plans by merging machine learning models with patient-specific data. These cutting-edge techniques, such deep learning and machine learning, will pave the way for customized therapy. This approach may also be used to promote collaboration between biologists, computer scientists, and doctors in order to create more efficient methods of arthritis diagnosis and treatment.

In the future, to promote research on the relationship between pathogenic microorganisms and arthritis, researchers will need to generate larger and more complete public datasets in order to better use machine learning and deep learning methodologies.

Robust methods for comprehending the intricate connection between pathogenic microorganisms and arthritis are offered by machine learning and deep learning methodologies. The advancement of these technologies offers fresh hope for the identification and management of arthritis, notwithstanding the difficulties. In the future, we anticipate seeing more creative ideas that will result in improved therapies for arthritis patients as multidisciplinary collaboration grows and technology progresses.

## Recognizing and categorizing infections

Researchers can reliably identify the bacteria that cause infectious arthritis from joint fluids and other clinical samples by using deep learning algorithms (Lo and Lai, 2023). These algorithms can differentiate between various bacteria and viruses by examining their gene sequences and patterns of protein expression, which serves as a foundation for creating specialized treatment plans (Choi et al., 2021). In clinical practice and medical research, precise pathogen identification and categorization is essential for infectious arthritis. Numerous bacteria and viruses can cause infectious arthritis, a dangerous condition for which a prompt and precise diagnosis is essential to the development of a successful treatment strategy. From joint fluids and other clinical samples, researchers can now reliably identify the bacteria and viruses that cause infectious arthritis thanks to the growing use of deep learning algorithms in bioinformatics and clinical diagnostics.

The novel applications of deep learning, a cutting-edge machine learning approach, include pathogen identification and classification, protein expression pattern recognition, and gene sequence analysis. Researchers can collect and analyze complicated biological data to pinpoint the precise species of germs that cause diseases by training deep neural networks. These algorithms are an effective diagnostic tool for infectious arthritis because they are particularly good at identifying patterns and characteristics from large volumes of data.

In the future, deep learning will be used in pathogen identification research in novel ways, with a focus on integrating additional kinds of biological data, optimizing algorithm performance, and increasing model interpretability. Additionally, as personalized medicine gains traction, deep learning methods will be used to customize treatment regimens based on the unique genetic make-up and clinical presentation of each patient (Zhang et al., 2018; Agarwal et al., 2020; Wason et al., 2020). Furthermore, improved management and treatment results for infectious arthritis will come from a closer integration of machine learning models with clinical decision support systems.

## Forecasting the course of an illness

A patient’s genetic makeup, way of life, and past infection history may all be analyzed by machine learning algorithms to determine their likelihood of having a particular kind of arthritis. These models reveal patterns and trends linked to the course of disease, which aids doctors in early diagnosis and intervention (Norgeot et al., 2019; Kedra et al., 2021).

## Discussion

The biomedical area has witnessed a tremendous advancement in machine learning and deep learning technologies, which has made them very promising for investigating the intricate connections between pathogenic microorganisms and orthopedic illnesses. Several studies have also shown how machine learning and deep learning can be used to predict the course of diseases, find links between particular pathogenic microorganisms and the emergence of orthopedic disorders, and develop novel approaches to illness prevention, diagnosis, and treatment.

It is our belief that the relationship between pathogenic microbes and orthopedic illnesses may be successfully shown by integrating cutting-edge techniques like machine learning and deep learning to assess microbiome data and clinical factors. Future risk and development of orthopedic disorders, including osteoarthritis, fracture infections, and spinal ailments, can be effectively predicted with the use of these technologies. Additionally, researchers have started to progressively uncover how these harmful bacteria impact the onset and progression of illnesses by examining the makeup of microorganisms and the interactions among hosts (Jansma and El Aidy, 2021; Zhou et al., 2022).

Future research should concentrate on the following areas to overcome these issues and progress the field: first, creating bigger, better-quality datasets that are more complete; second, enhancing data standards and sharing to make model training and validation easier. Second, the creation of fresh models and algorithms to enhance model interpretability and prediction accuracy, particularly when simulating intricate relationships between pathogenic microbes and their hosts. Furthermore, multidisciplinary cooperation—which calls for tight coordination between biologists, doctors, data scientists, and computer scientists—is essential to comprehending these intricate systems.

In addition to advancing our knowledge of pathogenic microbes and orthopedic disorders, the use of machine learning and deep learning technologies in these fields has the potential to completely transform clinical diagnoses and therapeutic approaches. For instance, doctors may monitor treatment outcomes, apply individualized therapies, and detect high-risk patients sooner thanks to the knowledge these technologies give. These technologies can also aid in the development of novel therapeutic approaches, such the targeting of certain pathogenic microorganisms or the modification of host-microbe interactions in the prevention or treatment of orthopedic illnesses.

In conclusion, there is a lot of promise and difficulty in the rapidly developing field of applying machine learning and deep learning to the study of pathogenic microorganisms and orthopedic illnesses. Through the removal of current obstacles and ongoing advancements in this field of study, we seek to provide new opportunities for the diagnosis, treatment, and prevention of orthopedic disorders as well as offer patients better access to healthcare.

In the study of pathogenic microorganisms in orthopedic diseases, machine learning and deep learning technologies offer new research directions and trends. These are primarily reflected in the areas of microbial community analysis, personalized healthcare, pathogen resistance prediction, fast and accurate pathogen identification and classification, and monitoring and outbreak prediction. Individual susceptibility to specific pathogenic microbial infections can be predicted by combining machine learning and deep learning techniques to analyze genetic data, lifestyle, and environmental factors of patients. This data can then be used to support personalized prevention and treatment plans. In the study of pathogenic microorganisms in orthopedic illnesses, machine learning and deep learning technologies have demonstrated considerable promise. They aid in quick diagnosis, accurate treatment, and efficient prevention, all of which enhance patient outcomes and quality of life. These technologies will have more comprehensive and long-lasting uses in this industry as long as they continue to grow and improve.

## Conclusions

Specifically, deep learning and machine learning techniques are employed to comprehend and forecast the complex relationships between these microorganisms and orthopedic disorders. The role of pathogenic microorganisms in orthopedic illnesses is examined in this research. Through a review of the present literature, we can see that although deep learning and machine learning offer strong instruments for locating, categorizing, and forecasting the role of pathogenic microorganisms in orthopedic disorders, there are some challenges to be addressed. High-quality, standardized, and annotated biological data are currently hard to come by, which limits their application in model training and validation. Although machine learning and deep learning models are quite good at identifying patterns and predicting outcomes, interpretability is still a big problem. This is particularly true in the medical domain, where it is necessary for researchers and doctors to be able to comprehend the results made by models with ease. It can occasionally be challenging for a single machine learning or deep learning model to collect all the pertinent biological signals due to the complexities of pathogen-host interactions. Further study must look at more complex models and algorithms to accurately depict these complex biological processes.

## Author contributions

ZS: Conceptualization, Data curation, Formal analysis, Funding acquisition, Investigation, Methodology, Project administration, Resources, Software, Supervision, Validation, Visualization, Writing – original draft, Writing – review & editing. HG: Conceptualization, Data curation, Investigation, Software, Supervision, Writing – original draft. BW: Conceptualization, Investigation, Software, Visualization, Writing – original draft. SZ: Conceptualization, Data curation, Formal analysis, Investigation, Methodology, Project administration, Software, Supervision, Validation, Writing – original draft.
